# Screening depression and anxiety in Indigenous peoples: A global scoping review

**DOI:** 10.1177/13634615231187257

**Published:** 2023-07-25

**Authors:** Kathryn Meldrum, Ellaina Andersson, Torres Webb, Rachel Quigley, Edward Strivens, Sarah Russell

**Affiliations:** 1 104560 James Cook University; 2 557360 Monash Children's Hospital; 3 188689 Queensland Health, Cairns and Hinterland Hospital and Health Service

**Keywords:** depression, anxiety, Indigenous peoples, screening tools

## Abstract

Indigenous peoples’ worldviews are intricately interconnected and interrelated with their communities and the environments in which they live. Their worldviews also manifest in a holistic view of health and well-being, which contrasts with those of the dominant western biomedical model. However, screening depression and/or anxiety in Indigenous peoples often occurs using standard western tools. Understandably, the cultural appropriateness of these tools has been questioned. The purpose of this scoping review was to map the literature that used any type of tool to screen depression or anxiety in Indigenous adults globally. A systematic scoping review method was used to search databases including, but not limited to, CINAHL, PubMed, Scopus and Google. Database-specific search terms associated with Indigenous peoples, depression and anxiety, and screening tools were used to identify literature. In addition, citation searches of related systematic reviews and relevant websites were conducted. The data set was limited to English language publications since database inception. Fifty-four publications met the review's inclusion criteria. Most studies were completed in community settings using standard western depression and anxiety screening tools. Thirty-three different tools were identified, with the Patient Health Questionnaire-9 being the most frequently used. The review's findings are concerning given repeated calls for culturally appropriate screening tools to be used with Indigenous peoples. Although there has been some work to cross-culturally adapt depression screening tools for specific Indigenous populations, clearly more clinicians and researchers need to be aware of, and use, culturally appropriate approaches to screening.

## Introduction

The worldviews of Indigenous peoples are intricately interrelated and interconnected with those of their communities and the environments in which they live. Indigenous people conceptualise health and well-being more holistically (Gall et al., 2021) than the dominant western biomedical paradigm (Le Grande et al., 2017; Wilson & Richmond, 2009). Physical, mental, spiritual and psychosocial well-being are not discrete as they are in western conceptualisations of health (Wilson & Richmond, 2009).

For the purposes of this scoping review, the definition of an Indigenous person proposed by the United Nations (United Nations, 2013) has been adopted. Although the United Nations are of the view that a universal definition for Indigenous people is not necessary, they do identify that Indigenous people are

peoples in independent countries who are regarded as indigenous on account of their descent from the populations which inhabited the country, or a geographical region to which the country belongs, at the time of conquest or colonization or the establishment of present state boundaries and irrespective of their legal status retain social, cultural, economic, and political characteristics that are distinct from those of the dominant societies in which they live (United Nations, n.d., p. 5).

Screening for mental health disorders such as depression and anxiety conducted in Indigenous populations has typically used western (hereafter referred to as standard) tools. However, several authors (Black et al., 2015; Gomez Cardona et al., 2021; Gray et al., 2016; Janca et al., 2015; Le Grande et al., 2017; Shore & Manson, 1981; Verney et al., 2008) have suggested that standard screening tools are inappropriate owing to the different cultural conceptualisations of mental health and well-being and language used. Tests may measure different constructs in other populations (Reynolds & Suzuki, 2012) and Gone and Kirmayer (2020, p. 235) argued that adaptation of ‘mental health theories, models and interventions’ is necessary to support the mental health of Indigenous peoples.

Bias and equivalence are two important methodological concepts that need to be attended to in cross-cultural research (He & van de Vijver, 2012). Statistically, bias is defined as a systematic error in the estimation of value (Reynolds & Suzuki, 2012). Bias can be found in the underlying construct being measured and its consequent items (questions) and the method (sampling, instrument and administration) (He & van de Vijver, 2012). Therefore, tests designed for and validated with the majority population as participants may measure different constructs in a minority population (Reynolds & Suzuki, 2012), whereas equivalence is related to cross-cultural comparisons. For example, where comparisons between Indigenous Australian and Anglo-Celt participants have been conducted (Davis et al., 2015), the tool used to screen for depression, in this case the Patient Health Questionnaire (PHQ-9), should lead to equivalent comparisons between the two groups. Both bias and equivalence have important implications for studies investigating depression and/or anxiety with Indigenous populations. In the example above, bias is present in the screening tool used with the Indigenous population because it was developed in the United States using predominantly White participants (Kroenke et al., 2001). Concomitantly, equivalence cannot be achieved. Equitable access to mental health support for people with depression and/or anxiety relies first on screening using a valid and reliable tool (instrument or scale). Ensuring that tools are equivalent and unbiased is the first step to achieving this aim.

This scoping review is situated in the context of a broader research project designed to develop a culturally appropriate depression and anxiety screening tool for Aboriginal and Torres Strait Islander communities in Far North Queensland, Australia. This project seeks to address the difficulties experienced by the research team (Russell et al., 2021, 2022) when using standard depression and anxiety screening tools in the Torres Strait. Issues emerged with the unfamiliar words and concepts used that resulted, at times, in questions being misinterpreted or viewed as offensive. This situation had the potential to impair the therapeutic rapport that the clinicians/researchers were trying to establish with participants. Consequently, the focus of this scoping review was to map the extant literature related to screening for depression and anxiety in Indigenous populations globally to inform this wider project. A scoping review was the most appropriate systematic approach because it permits mapping of published and grey literature (Arksey & O’Malley, 2005), including the approaches of various governments to obtaining mental health information from population-based surveys.

Preliminary searches of relevant databases indicated that there were no scoping reviews currently underway on this topic. Although several systematic reviews related to similar topics such as cultural concepts of distress (Kohrt et al., 2014) and mental health in Indigenous populations (Black et al., 2017, 2015; Bowen et al., 2014; Chan et al., 2021; Dingwall & Cairney, 2010; Jorm et al., 2012; Kisely et al., 2017; Nelson & Wilson, 2017) have been conducted, they are limited to specific Indigenous populations or cohorts within them. Consequently, the objective of this scoping review was to more broadly assess the extent of the literature related to tools used for screening depression and anxiety in Indigenous adults globally.

## Method

The protocol for scoping reviews outlined by the Joanna Briggs Institute (Peters et al., 2020) was used to guide data collection. The scoping review was registered with The Open Science Framework database (https://osf.io/9azwd). In line with the recommendations of Peters et al. (2020), this scoping review did not undertake a critical appraisal of individual sources because the aim was to gather as much information as possible from both the published and grey literature, some of which was obtained from national health surveys of Indigenous peoples in which the methods of obtaining the information was difficult to ascertain. In addition, the focus of the review was to scope the range of tools used to screen depression and anxiety of Indigenous peoples, rather than the methods used to develop or implement such tools. Because this scoping review collected data from open sources ethical approval was not required.

Data was collected in three phases. Phase one focused on search strategy, phase two on data selection and phase three on data extraction. The research question that guided data collection was: What tools are used to screen depression and anxiety in Indigenous adults globally? Associated sub-questions that guided inclusion criteria included:

1. In what geographical, cultural, clinical or other contexts is screening for depression and anxiety in Indigenous adults taking place?
2. Why are depression and anxiety screening tools being used?
3. Are they valid and reliable for the population that they are being used with?

### Inclusion criteria

The Participant, Concept, Context framework was used to develop the inclusion criteria and search strategy employed during the data collection phase of the review. Studies whose participants were Indigenous adults (≥18 years) from any part of the world were included.

The concepts that framed the inclusion criteria included any tools used to screen for depression and anxiety in Indigenous peoples, whether or not they were specifically designed for this population. Published and unpublished/grey literature that specifically identified Indigenous participants who met the definition of Indigenous adopted for this scoping review were included in the data set. In addition to retaining distinct characteristics that are different from the dominant society (United Nations, n.d.), Article 33 of the United Nations Declaration on the Rights of Indigenous peoples stated that the right to identify as an Indigenous person (self-identification) is fundamental (United Nations, 2007). Consequently, published and unpublished literature in which participants were identified as Indigenous or where identifying titles were used such as, but not limited to, First Nations, Māori, Native American were included in the scoping review. For example, participants in Sorlie et al. (2018) study came from ‘Sami core, Sami affiliation, Sami background or majority Norwegian groups’ (p. 1). According to Northern Norway (n.d.) the Sami peoples inhabit Norway, Sweden, Finland and Russia, and have distinct languages that are unrelated to Norwegian and other Indo-European languages. Traditionally, they herded reindeer and are recognised as the oldest culture in large areas of northern Norway. The Norwegian Sami were subject to a rigorous assimilation policy between 1860 and 1960 (Sorlie et al., 2018) and are a minority in the Norwegian population. In this scoping review, the term Indigenous is respectfully used to be inclusive of global populations who identify as such and are distinct from the dominant population.

In addition, studies that compared participants from non-Indigenous cultures with Indigenous participants, and those that used tools to describe, identify issues, determine the psychometric properties of (including validation), or determine prevalence of depression and/or anxiety of Indigenous adults, were included in the data set. Inclusion criteria in order of priority were:

- literature focused on depression and anxiety screening tools used with Indigenous adult populations;
- a specific tool used for screening depression and anxiety was identified;
- literature used primary data sources;
- tools were used with participants recruited from any setting (acute, primary healthcare, community);
- the publication constituted a complete paper or report (not an abstract, executive summary, editorial, etc.);
- only literature published in English was included.

The inclusion of data from primary data sources was chosen because the rationale for this scoping review is based on not only the use of depression and anxiety screening tools, but also the participant and researcher experience of interacting with the tool; for example, limitations to participation such as: Does written English literacy limit a participant's ability to self-report? Do western conceptualisations of depression and anxiety mean that questions in a screening tool are using words that are unfamiliar or do not relate to an Indigenous person's way of thinking and/or feeling about their mood? In addition, when the method for collecting the data is not described in detail, unintended limitations may not be able to be determined by the reader. Finally, often authors report limitations or difficulties that their participants had with interacting with the tool, or researchers found with tool administration, in the discussion section. Articles reporting the findings of secondary data analysis do not have this information to share in the discussion.

### Search strategy

The search strategy located both published and unpublished studies and reports. The databases searched included CINAHL, Emcare, Medline (Ovid), PsychInfo, PubMed (Ovid) and Scopus. Google, Google Scholar and website searches were used to locate sources of unpublished studies and grey literature. In addition, citation searches of all included sources of evidence were screened for additional studies. The reference lists of systematic reviews on similar topics were also searched for appropriate papers, reports or other data sources. Unpublished studies and reports, once located, were downloaded if that was possible. If it was not possible a screenshot of the information was taken, or the information was copied and pasted into a Word document. These documents were subsequently saved with all the other data. In addition, if a reference was available (through Google Scholar, for example) it was downloaded and imported into EndNote. If a citation was not available, the capture reference function available through EndNote was used and the citation was directly imported into EndNote Online. The full search strategies for all databases are provided in the online Supplemental material. Data were collected between August and September 2022.

### Data selection

**Figure 1.**
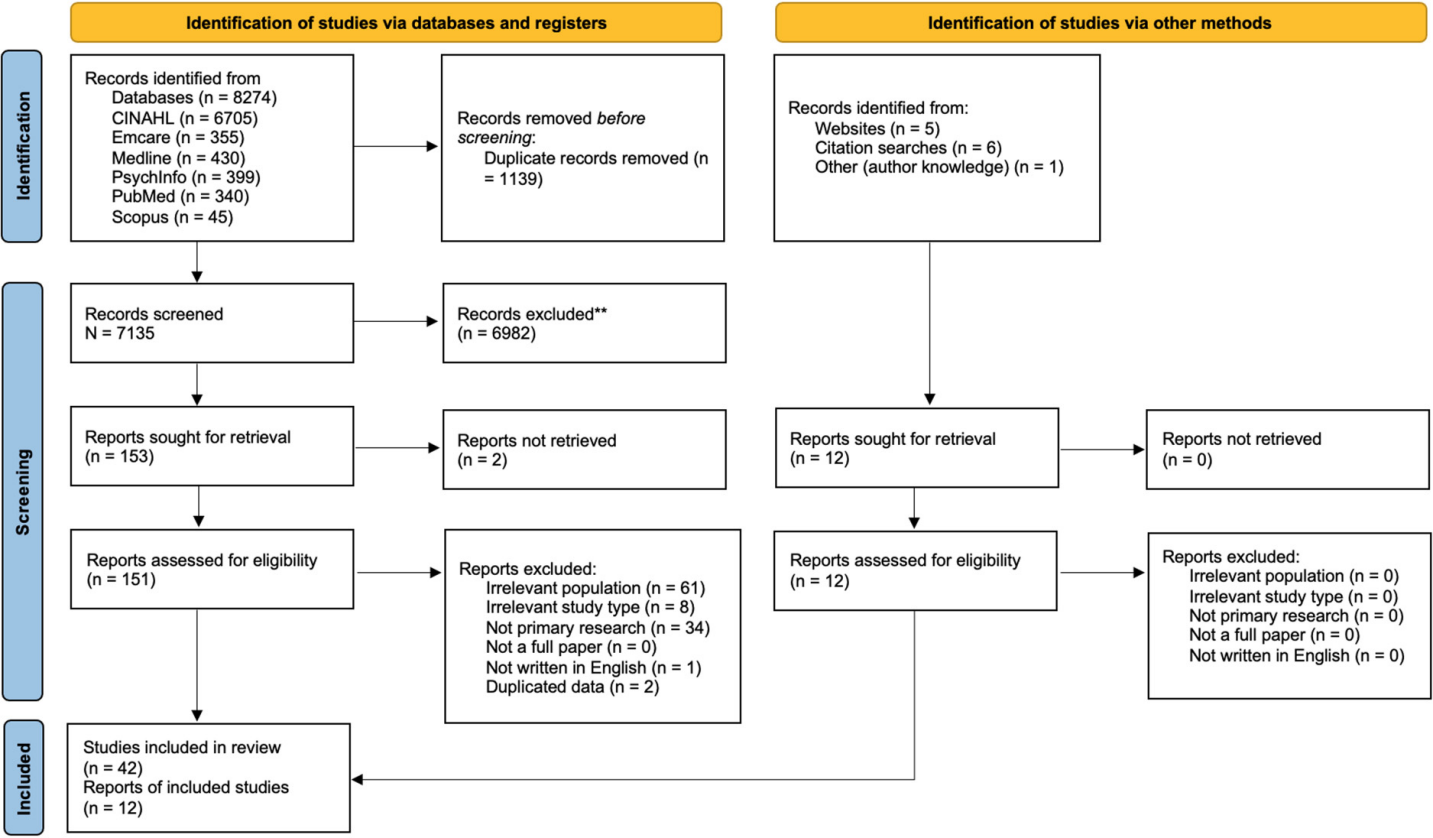
PRISMA study flowchart (adapted from Page et al., 2021).

Phase two of data collection was managed in EndNote (version 20.1; Clarivate Analytics) according to the method proposed by Peters (2017). Subsequently, a pilot test of the data collected using the search strategy (Peters et al., 2020) was conducted between the first two authors. An interrater reliability of 95% was achieved in the pilot test and discrepancies were discussed. This discussion facilitated further refinement of the inclusion criteria. The rest of the data collection process, including retrieval of full-text articles, website and citation searches, was conducted by the first author. Relevant citation information of all included literature was then entered into an Excel spreadsheet, where all subsequent screening took place. The second author used this database to independently screen full-text articles and reports. Any discrepancies were resolved through discussion. The data collection process is presented in a Preferred Reporting Items for Systematic Reviews and Meta-analyses extension for scoping review (PRISMA-ScR) flow diagram (2020) (Figure 1).

### Data extraction

Data was extracted independently by the first two authors and entered into an Excel spreadsheet. Aside from citation information, other data fields included: the country where the study was conducted; context; study population and sample size (*n*); population mean age (and/or range); study aim/purpose; method; tool type; study outcome; and key findings as they related to the research question and sub-questions. The tool type categories were: (1) unmodified standard (western) tool; (2) language translation of a standard tool; (3) cross-culturally adapted standard tool; and (4) non-western country-specific tool.

The spreadsheet was iteratively refined by the first two authors through discussion of the emerging themes as data extraction progressed. For example, tool type had two additional categories added (2 and 4) after data extraction commenced in response to emerging themes. Tool type 4 was included to enable the categorisation of an anxiety and depression tool developed specifically for the Chakma and Marma population living in Bangladesh. This tool type was different from tool type 1 (standardised western tools) because it was developed in a non-western country.

### Data analysis

Data was analysed quantitatively to identify geographic distribution and by context and date. Quantitative sums of tool types used were categorised according to the definitions provided above. Qualitative summing of the names of different types of tools and their related context was also conducted. Qualitative coding to develop themes focused on the rationale for tool use as well as its psychometric properties, limitations of the study and/or recommendations for future investigation was also conducted. The results are presented below.

## Results

This scoping review focused on answering the research question: What tools are used to screen for depression and anxiety in Indigenous adults globally?

### Yield of databases and other searches

Database searches yielded 8274 records, which were reduced to 7139 after duplicates were removed. Screening subsequently reduced the data set to 153 records. Full-text screening brought this number down to a total of 42 records, which were included in the final data set. While reviewing full-text records, a list of cited websites was made. This list was used to identify possible additional sources available on websites. Records obtained from searching the reference lists of all included papers, as well as systematic reviews on a related topic, yielded an additional six records. A search of websites including Google, Google Scholar and other relevant sites, for example the Canadian First Nations Information Governance Centre (https://fnigc.ca/) and the Australian Bureau of Statistics yielded a further five records. One further record known to the authors but not located through any database or other search was also included. The alternate search strategy yielded 12 records. All search strategies located a final data set of 54 records that met the inclusion criteria for the scoping review (Figure 1 and Table 1). Abbreviations for scales included in the studies listed in Table 1 are included at the end of the table. In addition, scales used to validate adapted and/or new scales appear in parentheses after the scale abbreviation.

**Table 1.** Characteristics of included records.

Author(s)	Year	Country of origin	Context	Tool type	Tool used (validation tool)
Lee et al.	2022	Canada	C	1	GAD-2; PHQ-2
Russell et al.	2022	Australia	C	1, 3	GAI, KICA-Dep
Danyluck et al.	2021	United States	C	1	CES-D (revised)
Faruk et al.	2021	Bangladesh	C	5	Anxiety scale; Depression scale
Wong et al.	2021	Australia	C	3	KICA-Dep
Gallardo-Peralta et al.	2020	Chile	C	1	GDS-15
Hsieh et al.	2020	United States	C	1	CES-D
Australian Bureau of Statistics (ABS)	2019	Australia	C	1	K5
Center for Disease Control (CDC)	2019–22	United States	C	1	Adapted K6
Easton et al.	2019	United States	C	1	PHQ-9
Getting It Right Collaborative	2019	Australia	PH	2	Adapted PHQ-9 (*MINI*)
Gray et al.	2019	United States	PH	1	BDI, CES-D, Tri-ethnic Center Depression Scale, SCL-90-R
Ing et al.	2019	United States	C	1	CES-D (short)
Tu et al.	2019	Canada	PH	1	PHQ-9
Walker et al.	2019	Panamá	PH, C	1	PHQ-9; K6
First Nations Data Governance Centre (FNDGC)	2018	Canada	C	1	K10
Shen et al.	2018	Australia	C	3	aPHQ-9
Sorlie et al.	2018	Norway	C	1	HSCL-10
Taylor et al.	2017	Australia	C	2	PHQ-9
Warne et al.	2017	United States	C	1	PHQ-2, GAD
Benoit et al.	2016	Canada	C	1	CES-D
Gachupin et al.	2016	United States	C	1	GDS-15; HAS
Gray et al.	2016	United States	C, PH	1	BDI, BAI, CES-D SCL-90-R
Walls et al.	2016	United States	C	1	MHC-SF, PHQ-9, BAI, PWB, CESD-10, MINI
Williamson et al.	2016	Australia	PH	1	K10
Davis et al.,	2015	Australia	C	1	PHQ-9
Firestone et al.	2015	Canada	C	1	K10
Gallardo-Peralta et al.	2015	Chile	C	1	GDS
Kading et al.	2015	United States	C	1	PHQ-9
Montag et al.	2015	United States	PH	1	PHQ-9
Roh et al.	2015	United States	C	1	GDS
Almeida et al.	2014	Australia	C	3	KICA-Dep
Isaacs et al.	2014	Australia	C	1	K10
ABS	2013	Australia	C	1	K5
Evans-Campbell et al.	2012	United States	C	1	MINI, CESD-10
Esler et al.	2008	Australia	PH	3	aPHQ-9
Froese et al.	2008	United States	C	1	PHQ-9
Letiecq et al.	2008	United States	C	1	CES-D
MacMillan et al.	2008	Canada	C	1	CIDI
Sahota et al.	2008	United States	C	1	PHQ-9
Verney et al.	2008	United States	C	1	CIDI
Whitbeck et al.	2006	United States and Canada	C	1	CIDI
ABS	2006	Australia	C	1	K5
Bell et al.	2005	United States	C		CES-D (EPESE version)
The MaGPIe Research Group	2005	Aotearoa	C	1	CIDI
Daniel et al.	2004	Canada	C	1	BSD
Duran et al.	2004	United States	PH	1	GHQ-12
De Coteau et al.	2003	United States	C	1	BAI, ASI
Whitbeck et al.	2002	United States	C	1	CES-D
Kaholokula et al.	1999	United States	C	1	CES-D
Goldwasser & Badger	1989	United States	PH	1	GHQ
Wilson et al.	1995	United States	PH	1	IDD
Gregory	1994	United States	PH	3	SDS
Westermeyer & Neider	1984	United States	NR	1	SDS

ASI: Anxiety Sensitivity Index; BAI: Beck Anxiety Inventory; BDI: Beck Depression Inventory; CESD-10 or CES-D: Center for Epidemiological Studies Depression Scale; CIDI: Composite International Diagnostic Interview; EPESE: Established Populations for Epidemiological Studies of the Elderly; GAD: Generalised Anxiety Disorder; GAI: Geriatric Anxiety Inventory; GDS-15: Geriatric Depression Scale; GHQ-12: General Health Questionnaire; HAS: Hamilton Anxiety Scale; HSCL-10/25: Hopkins Symptom Checklist; IDD: Inventory for Diagnosing Depression; K10, 6 or 5: Kessler 10; KICA-Dep: Kimberly Indigenous Cognitive Assessment – Depression; MHC-SF: Mental Health Continuum-Short Form; MINI: MINI-International Neuropsychiatric Interview; PHQ: Patient Health Questionnaire; PWB: Ryff's Psychological Well-being; SCL-90-R: Symptom Checklist-90-Revised; SDS: Zung Self-rated Depression Scale.

A: acute; C: community; NR: not reported; PH: primary healthcare.

### Geographical context

**Figure 2.**
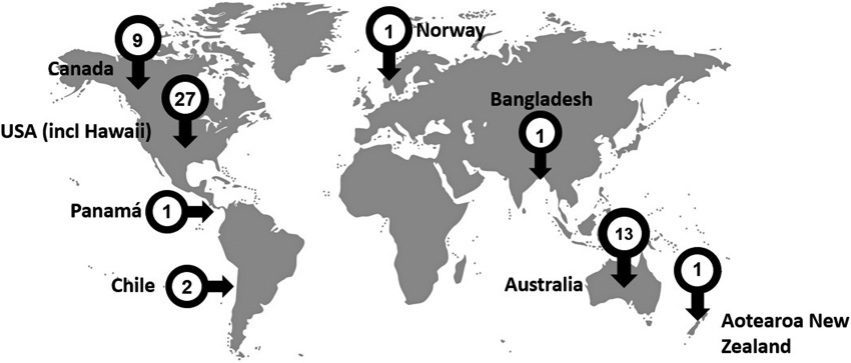
Spread of studies across the globe.

Twenty-seven records were retrieved from studies conducted in the United States, which was the largest number of records sourced from one country. Thirteen studies were conducted in Australia and nine in Canada, with one of these studies being conducted and counted in the totals for both Canada and the United States. Two studies were conducted in Chile, and one each in Aotearoa (New Zealand), Bangladesh, Norway and Panamá. Figure 2 illustrates the spread of the studies globally.

In line with the geographical context in which studies took place, most were conducted with Indigenous peoples who identified as American Indian and/or Alaskan Native and/or Native Hawai’ian. Australian Aboriginal and/or Torres Strait Islanders peoples and Canadian First Nations, Metis and Inuit peoples participated in 13 and 9 studies, respectively. Smaller numbers of studies that met the inclusion criteria for this scoping review were conducted with the Aymara and Mapuche from Chile (*n* = 2), the Māori from Aotearoa, (*n* = 1), the Chakma and Marma from Bangladesh (*n* = 1), the Sämi from Norway (*n* = 1) and the Kuna Indians from Panamá (*n* = 1).

### Study contexts

**Figure 3.**
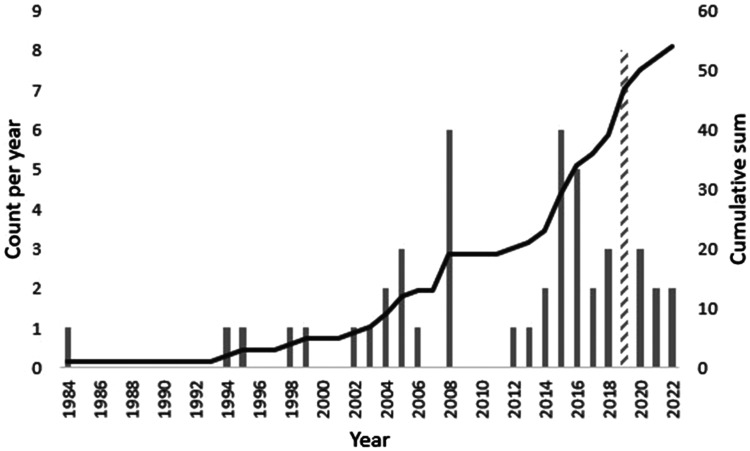
Publication dates of the included studies.

Publication year and study setting were examined as part of the study context for the included studies. Only five papers (9%) published before 2000 met the inclusion criteria. Between 2000 and 2010, 14 (25%) papers that met the inclusion criteria for this scoping review were published. By contrast, between 2010 and 2022, 35 (65%) papers meeting the inclusion criteria were published. The greatest number of studies (*n* = 8, 15%) were published in 2019. However, six (11%) studies were published in 2008, which was the largest annual number for the decade 2000–2010. This shows an increased trend in screening for depression and anxiety in Indigenous populations and sharing the findings through publication. Figure 3 illustrates the number of studies (count) per year as well as the cumulative count overall.

Most studies took place with participants recruited from their community (*n* = 43, 80%). Participants recruited from primary health contexts accounted for the remainder of the studies (*n* = 12, 22%). Two studies took place in both community and primary care settings, which is why the percentages above equal more than 100%. No studies took place in acute care settings. One study did not report its setting. Four studies were conducted with participants who identified as female (7%) and one (2%) with males. However, most studies (*n* = 49, 90%) took place with male and female participants. In 28 studies the mean age or age range of participants was above 40 years.

### Tool type

The depression and anxiety screening tools identified in the studies included in this scoping review were divided into four categories. Standard tools such as the PHQ-9, Kessler 10 (K10) and Center for Epidemiological Studies Depression Scale (CES-D) used in their unmodified form were included in category 1. For the purposes of this scoping review, standard tools are defined as those that have been developed in western countries and validated predominantly with people of an Anglo/European background. For example, the PHQ-9 was developed in the United States during the late 1990s and validated with participants, 79% of whom identified as White and 66% as female (Kroenke et al., 2001). Likewise, the CES-D (Radloff, 1977) was developed in the United States with a predominantly White population (Comstock & Helsing, 1977). A standard tool was used in most studies (*n* = 46, 85%) to screen for depression and anxiety in Indigenous peoples globally.

Category 2 included two studies that modified the standard tool by directly translating it into another language. For example, the PHQ-9 has been translated into more than 100 languages (Kroenke, 2021). Two studies (4%) included in this scoping review fitted into this category.

Adaptations of standard screening tools to make them more culturally appropriate (category 3) were used in six (11%) of studies. For example, Almeida et al. (2014) developed the Kimberly Indigenous Cognitive Assessment–Depression (KICA-Dep) by adapting the PHQ-9 for use with older Indigenous peoples from the Kimberly and Derby regions of Western Australia.

Finally, only one study used a depression and anxiety screening tool specifically designed for a non-western country (category 4). Faruk et al. (2021) used a depression scale and an anxiety scale specifically developed for the Chakma and Marma peoples of Bangladesh, to screen Indigenous peoples of the Chittagong Hill Tracts.

### Tools used

Thirty-three different tools were used in the studies included in the scoping review. They were grouped into two categories: screeners and intermediaries. Screening tools are designed to identify people exhibiting signs of disturbances in their mental well-being who need to be referred to a doctor or psychiatrist for diagnosis and treatment (Cairney et al., 2007; Roberts & Vernon, 1983). They are designed to be used by a range of health professionals and take a short time to complete and are consequently economical (Roberts & Vernon, 1983). In contrast, intermediaries such as the Composite International Diagnostic Interview (CIDI) and MINI-International Neuropsychiatric Interview (MINI) take longer to complete and must be administered by a trained individual who is familiar with the Diagnostic and Statistical Manual of Mental Disorders, Fifth Edition (DSM-5) classification and diagnostic criteria. However, because they do not have to be administered by a psychiatrist, they are suitable for use in community and primary healthcare settings. Table 2 presents the three most used screening and intermediary tools and their adaptations.

**Table 2.** Top three tools used to screen depression and anxiety in Indigenous populations.

Type	Tool	Prevalence of use	Total
**Screening**			
	PHQ9		18
	Standard	10	
	Adapted	3	
	KICA-Dep	3	
	PHQ-2	2	
	CES-D		12
	Standard	8	
	CESD-10	2	
	CES-D (EPESE version)	1	
	CESD-R	1	
	Kessler		9
	K10	4	
	K6	1	
	K5	4	
**Intermediary**			
	CIDI		5
	MINI		2

CESD-10 or CES-D: Center for Epidemiological Studies Depression Scale; CIDI: Composite International Diagnostic Interview; EPESE: Established Populations for Epidemiological Studies of the Elderly; K10, 6 or 5: Kessler Psychological Distress Scale (10, 6, 5 question versions); KICA-Dep: Kimberly Indigenous Cognitive Assessment – Depression; MINI: MINI-International Neuropsychiatric Interview; PHQ: Patient Health Questionnaire.

The standard tool used most often was the PHQ-9 (18 times). In its standard form, this tool was more commonly used for clinical studies determining the interactions between depression and diabetes (Davis et al., 2015; Sahota et al., 2008; Taylor et al., 2017) and sleep (Froese et al., 2008). It was also used to evaluate clinical interventions (Montag et al., 2015; Tu et al., 2019), examine distal and proximal factors related to depression and population prevalence in an Indigenous population in Panamá (Walker et al., 2019) and in the United States (Kading et al., 2015; Walls et al., 2016). Adapted forms of the PHQ-9 have been used with Australian Aboriginal and Torres Strait Islander populations (Almeida et al., 2014; Esler et al., 2008; Getting It Right Collaborative, 2019; Russell et al., 2022; Shen et al., 2018; Wong et al., 2021).

The CES-D was the second most frequently used standard tool (12 times). This screening tool was used in eight studies in its standard form and four in an adapted form. The CES-D (and its adapted forms) was used almost exclusively in studies conducted with community samples investigating the correlation between depression and other lifestyle (Benoit et al., 2016; Evans-Campbell et al., 2012; Kaholokula et al., 1999) and/or environmental/social factors (Hsieh et al., 2020; Ing et al., 2019; Letiecq et al., 2008; Roh et al., 2015; Whitbeck et al., 2002). Depression prevalence was also determined using the CES-D (Bell et al., 2005) as well as the psychometric properties of other scales using the CES-D as a validator (Gray et al., 2019). Finally, Walls et al. (2016) used both the PHQ-9 and CES-D to describe the mental well-being of American Indians.

The Kessler screening tools (K10, K6, K5) were the third most commonly used tool; being cited nine times. They have been used in Indigenous population-based screens (ABS, 2006, 2019; CDC, 2019–2022; FNDGC, 2018), as well as more general studies (Firestone et al., 2015; Isaacs & Lampitt, 2014; Williamson et al., 2016). Walker et al. (2019) also used the K6 to measure serious psychological distress with the PHQ-9 in a primary health and community sample in Panamá.

Intermediary tools such as the CIDI and the MINI have been used five and two times, respectively. Both tools have been used as a stand-alone (MacMillan et al., 2008; The MAGPIe Group, 2005; Whitbeck et al., 2006) or with other screening tools (Evans-Campbell et al., 2012; Walls et al., 2016).

Some studies used more than one tool, especially when validating adapted tools. For example, the Getting It Right Collaborative (2019) validated Brown et al. (2016) adapted PHQ-9 against a MINI. Gray et al. (2016, 2019) used the Beck Anxiety Inventory (BAI), Beck Depression Inventory (BDI), CES-D and Tri-Ethnic Depression Scale to evaluate psychometric properties of these scales for Northern Plains Indians.

### Reason for tool use

Tools used to screen depression and anxiety were categorised into nine reasons for use: determining prevalence; assessing the psychometric properties (including validation); population comparison; tool development; identifying of mental health needs; evaluating interventions; generating new knowledge; establishing the relationship of depression with (an)other variable(s); and establishing the relationship of anxiety with (an)other variable(s).

The greatest use of depression screening tools has been to determine the correlation between it and other lifestyle and/or environmental and/or social factors. Twenty-nine studies (54%) were categorised by this use. The second highest use (*n* = 15, 28%) was to determine the prevalence of depression and/or anxiety in Indigenous peoples. Often, these studies were responding to the observation that Indigenous peoples experience increased mental health support needs, and therefore were looking to establish prevalence rates. Comparing depression and/or anxiety in different population groups (*n* = 8, 15%) was the third highest reason for use. Assessing the psychometric properties (including validation) (*n* = 5, 9%) of tools was the fourth highest reason for use, whereas generating new knowledge (*n* = 3, 5%) was fifth. Fewer studies used screening tools to identify mental health needs (*n* = 2, 4%), develop new tools (*n* = 2, 4%), evaluate an intervention (*n* = 2, 4%) and determine the correlation between anxiety and other lifestyle and/or environmental and/or social factors (*n* = 1, 2%). Some studies were focused on more than one reason for using a depression and/or anxiety screening tool, consequently the totals are greater than total number of studies in the data set and percentages are greater than 100.

### Reliability and validity

The overarching quality of a screening tool is determined by its reliability and validity for the population it is being used with (Alarcón et al., 2002; Sorlie et al., 2018). Given its importance, surprisingly few authors determined tool reliability (*n* = 16, 30%) and even fewer validity (*n* = 6, 11%). However, more authors measured reliability as opposed to assuming it (*n* = 5, 9%) based on the findings of other studies. In contrast, more authors assumed tool validity (*n* = 10, 19%) rather than measuring it in their sample. The number of studies that did not address tool reliability (*n* = 33, 61%) or validity (*n* = 37, 69%) is a significant limitation given that the majority used standard tools not originally developed for Indigenous populations.

## Discussion

The findings of this scoping review highlight that standard tools, the PHQ-9, CES-D and the Kessler Psychological Distress Scale (and their respective adaptations), remain the most frequently used, despite calls for mental health screening of Indigenous peoples to be achieved using culturally appropriate tools (Adams et al., 2014; Brinckley et al., 2021; Gomez Cardona et al., 2021; Le Grande et al., 2017). The findings of this scoping review also respond to a recent call to critically assess mental health theories, models and interventions for, and with, Indigenous peoples (Gone & Kirmayer, 2020), by providing evidence of current approaches to screening for depression and anxiety.

### Contexts for the use of depression and/or anxiety screening tools

Half of the data set for this scoping review contained studies conducted in the United States (including Hawai’i). These studies were predominantly conducted with participants recruited from the community and were more likely to be investigating the relationship of depression with lifestyle, environmental or social variables. The second highest number of studies came from Australia. Similarly, Australian studies were predominantly conducted with participants recruited from community settings and also investigated the relationship of depression with other variables. However, proportionally more studies in Australia were focused on validation or adaptation/new screening tool development when compared with the United States. No tool adaptation for Indigenous peoples took place in the United States and only two studies that met the inclusion criteria for this scoping review focused specifically on validation (Gray et al., 2016, 2019). Gray et al. (2016, 2019) also validated depression and anxiety screening tools [BDI, BAI, CES-D, Tri-ethnic Centre Depression Scale, Symptom Checklist-90-Revised (SCL-90-R)] that, according to the findings of this scoping review, are less commonly used. By contrast, Australian validation and new tool development studies were focused on adapting the most used tool – the PHQ-9 – for use with Australian Indigenous peoples.

### Cross-cultural adaptation of standard tools

As stated previously, the quality of screening tools is dependent on their reliability and validity. Australian studies that met the inclusion criteria for this scoping review focused on cross-cultural adaptation of the PHQ-9. Rationales for tool adaptation have been focused on cultural acceptability, which includes ensuring cultural relevance and revising the wording of each item. For example, the Getting It Right Collaborative (2019) validated the previously adapted PHQ-9 (Brown et al., 2016, 2012) for use with Aboriginal and Torres Strait Islanders living in urban, rural and remote Australia. When reviewing adaptations made to the PHQ-9 by Brown et al. (2012, 2016), the Getting It Right Collaborative noted that many key symptoms of western conceptualisations of depression were not present in the Central Desert Aboriginal men that participated in Brown et al.’s (2012, 2016) studies. By contrast, Aboriginal men reported ‘anger, weakened spirit, irritability, excessive worry, rumination, and drug or alcohol use’ (Getting It Right Collaborative, 2019, p. 24). To make the PHQ-9 culturally relevant these symptoms needed to be reflected in the adapted PHQ-9. In addition, the construct of hopelessness was omitted because participants felt that their perceptions of depressed mood encapsulated it (Brown et al., 2012).

Making the PHQ-9 culturally relevant for Australian Aboriginal people has also meant that additional items have been included in the screener. For example, Almeida et al. (2014) split the item about suicidal ideation making their KICA-Dep adaptation of the PHQ-9 an 11-item screener. In addition, in the precursor to the publication included in this data set (Esler et al., 2008), Esler and colleagues included an item about anger in their modification of the PHQ-9 (Esler et al., 2007). Also, changes to word use in some items included using the word sad rather than depressed (Esler et al., 2008), and use of the words slack and shame (Almeida et al., 2014) for feelings of lethargy and guilt, respectively.

Finally, cross-cultural adaptation makes the screener more acceptable. This was highlighted by both Esler et al. (2008) and the Getting It Right Collaborative (2019) who stated that the adapted PHQ-9 screener that they used was acceptable to their Aboriginal and Torres Strait Islander participants. Consequently, tools that have undergone cross-cultural adaptation are more likely to be appropriate for the Indigenous population for which they have been validated compared with standard tools.

Standard tools may continue to be used because researchers and practitioners in this area do not have the resources to cross-culturally adapt or develop new tools for screening for depression and/or anxiety in Indigenous populations. As studies that have undertaken these activities have indicated, it is critical that Indigenous peoples are involved in this process (Brown et al., 2013; Carlin et al., 2019; Janca et al., 2015). Ideally, Indigenous people should lead the work so that both Indigenous conceptualisations of depression and anxiety, as well as data interpretation, are undertaken through a culturally appropriate lens (Chan et al., 2021). Therefore, key resource issues such as relationships with Indigenous peoples, cultural knowledge, and the associated time and money required to undertake this work may impact on the ability of some researchers and practitioners to either cross-culturally adapt and validate a standard tool or develop an entirely new one.

### Implications of the findings

The most used screening tool was the PHQ-9 and its adaptations. Although two adaptations have already been made for specific groups of Indigenous Australians, researchers should consider adapting the PHQ-9 either as a language translation or cross-cultural adaptation and validating it for their population as part of their research. We acknowledge that there are resourcing implications for projects when this takes place, but we argue that it is necessary because of the cultural inappropriateness of standard tools.

Many authors (Brinckley et al., 2021; Brown et al., 2013; Butler et al., 2007; Chan et al., 2021; Janca et al., 2015; Le Grande et al., 2017; Nelson & Wilson, 2017) have highlighted the inappropriateness of using standard tools to screen mood and anxiety in Indigenous peoples. Indigenous peoples conceptualise health and well-being holistically, which is interconnected and interrelated with the well-being of their communities and their land (Gall et al., 2021; Sørly et al., 2021; Wilson & Richmond, 2009). Consequently, the individualistic western model does not capture Indigenous holistic conceptualisations of well-being (Le Grande et al., 2017; Wilson & Richmond, 2009). In addition, Brown et al. (2013, p. 2) highlighted that ‘labelling Indigenous people with western diagnostic classifications without assessing their equivalence, relevance, acceptability or utility serves little purpose if such labels are devoid of the context and realities’ of their lives. Also, cultural understandings of word use are different (Brinckley et al., 2021; Brown et al., 2013; Chan et al., 2021; Snodgrass et al., 2017), as are expressions of distress (Kohrt et al., 2014; Snodgrass et al., 2017). Furthermore, questionnaires written in the English language that rely on written responses may be affected by literacy and education levels, as well as different conceptualisations of time and numbers (Janca et al., 2015). Hence, the findings of this review indicate that more work is required to support the needs of Indigenous peoples, starting with the development, validation and use of culturally appropriate screening tools.

### Findings related to the broader study which scoping review is situated

This scoping review identified one paper (Taylor et al., 2017) that used PHQ-9 to investigate the relationship between diabetes and depression in Torres Strait Islanders living in the Torres Strait. The authors acknowledged that the PHQ-9 was not validated for use with the population. Another paper (Russell et al., 2022) identified that the Geriatric Anxiety Inventory (GAI) and KICA-Dep were also culturally inappropriate for use with Torres Strait Islanders living in the Torres Strait. This scoping review therefore presents evidence for the importance of cross-culturally adapting or creating a new tool/approach to screen depression and anxiety (the mental well-being) of people living in the Torres Strait or the Northern Peninsula Area of Australia.

### Limitations of the scoping review

Including only English language publications was a limitation of this study, because we may have missed studies in other populations globally. For example, there are a limited number of studies from the countries of South America included in this scoping review, which may have been published in other languages such as Spanish. Therefore, collaborating with researchers who speak other languages could have enhanced the global reach of the scoping review.

## Conclusion

This scoping review gathered evidence of the tools used to screen depression and/or anxiety of Indigenous peoples globally. The overarching finding indicates that standard tools developed using the western biomedical model of mental health predominate when screening depression and anxiety in Indigenous peoples. However, the cross-cultural adaptation and validation of standard tools has begun to emerge in the past decade, with Australian researchers leading this work. This is important given identified issues with the cultural appropriateness of standard tools used with Indigenous peoples including: holistic conceptualisations of health and well-being, cultural expressions of distress, word use and written English language literacy.

Implications of the scoping review findings indicate that national governments, researchers and clinicians should review their approach to screening depression and anxiety in Indigenous peoples. The PHQ-9 has been adapted in Australia. Population-based screening of Indigenous peoples by governments using standard tools such as the K10 and, in Australia, the K5, would benefit from cross-cultural adaptation. This work has already been done in Australia by Brinckley et al. (2021) – not included in the data set because participants included people <18 years – which could be used as a model by other governments.

Opportunities for future research in this area are provided by our narrow approach to selecting the inclusion criteria for this scoping review. The inclusion criteria selected excluded publications that did not provide an outcome of the screening tool used with Indigenous peoples. For example, a prevalence of depression and/or anxiety disorders, the identification of issues, the outcomes of an intervention or comparison data. This choice excluded some studies that reported approaches to adapting culturally appropriate tools or developing new ones. In addition, the validity, specificity and sensitivity of tools were not reported in this scoping review. Therefore, systematic reviews of approaches to cross-cultural adaptation or the development of new tools, as well as their validity, specificity and sensitivity present potential avenues for further research.

## Supplemental Material

sj-pdf-1-tps-10.1177_13634615231187257 - Supplemental material for Screening depression and anxiety in Indigenous peoples: A global scoping review Supplemental material, sj-pdf-1-tps-10.1177_13634615231187257 for Screening depression and anxiety in Indigenous peoples: A global scoping review by Kathryn Meldrum, Ellaina Andersson, Torres Webb, Rachel Quigley, Edward Strivens and Sarah Russell in Transcultural Psychiatry

## References

[bibr1-13634615231187257] ABS. (2006). *National Aboriginal and Torres Strait Islander Health Survey, Australia 2004–05 (cat. no. 4715.0)* . Retrieved https://www.abs.gov.au/AUSSTATS/abs@.nsf/Lookup/4715.0Main+Features12004-05

[bibr2-13634615231187257] ABS. (2013). *Australian Aboriginal and Torres Strait Islander Health Survey: First Results, Australia, 2012–13* . Retrieved https://www.abs.gov.au/ausstats/abs@.nsf/mf/4727.0.55.001

[bibr3-13634615231187257] ABS. (2019). *National Aboriginal and Torres Strait Islander Health Survey* . Retrieved https://www.abs.gov.au/statistics/people/aboriginal-and-torres-strait-islander-peoples/national-aboriginal-and-torres-strait-islander-health-survey/latest-release

[bibr4-13634615231187257] Adams Y. Drew N. M. Walker R. (2014). Principles of practice in mental health assessment with Aboriginal Australians. In: Dudgeon P. Milroy H. Walker R. (Eds.), Working together: Aboriginal and Torres Strait Islander mental health and wellbeing principles and practice (2nd edn, pp. 271–288). Department of the Prime Minister and Cabinet.

[bibr5-13634615231187257] Alarcón R. D. Bell C. C. Kirmayer L. J. Lin K.-M. Üstün B. Wisner K. L. (2002). Beyond the funhouse mirrors: Research agenda on culture and psychiatric diagnosis. In: Kupfer D. J. First M. B. Reigier D. A. (Eds.), A research agenda for DSM-V (pp. 219–281). American Psychiatric Association.

[bibr6-13634615231187257] Almeida O. P. Flicker L. Fenner S. Smith K. Hyde Z. Atkinson D. Skeaf L. Malay R. LoGiudice D. (2014). The Kimberley assessment of depression of older Indigenous Australians: Prevalence of depressive disorders, risk factors and validation of the KICA-dep scale. PLoS ONE, 9(4), e94983. 10.1371/journal.pone.0094983 PMC3989269 24740098

[bibr7-13634615231187257] Arksey H. O’Malley L. (2005). Scoping studies: Towards a methodological framework . International Journal of Social Research Methodology, 8(1), 19–32. 10.1080/1364557032000119616

[bibr8-13634615231187257] Bell R. A. Smith S. L. Arcury T. A. Snively B. M. Stafford J. M. Quandt S. A. (2005). Prevalence and correlates of depressive symptoms among rural older African Americans, Native Americans, and whites with diabetes. Diabetes Care, 28(4), 823–829. Retrieved http://ovidsp.ovid.com/ovidweb.cgi?T=JS&PAGE=reference&D=med6&NEWS=N&AN=15793180 10.2337/diacare.28.4.823 15793180 PMC1592640

[bibr9-13634615231187257] Benoit A. C. Cotnam J. Raboud J. Greene S. Beaver K. Zoccole A. O’Brien-Teengs D. Balfour L. Wu W. Loutfy M. (2016). Experiences of chronic stress and mental health concerns among urban Indigenous women. Archives of Women's Mental Health, 19(5), 809–823. https://doi.org/https://dx.https://doi.org/10.1007/s00737-016-0622-8 10.1007/s00737-016-0622-8 26961003

[bibr10-13634615231187257] Black E. B. Kisely S. Alichniewicz K. Toombs M. (2017). Mood and anxiety disorders in Australia and New Zealand's indigenous populations: A systematic review and meta-analysis. Psychiatry Research, 255, 128–138. 10.1016/j.psychres.2017.05.015 28544944

[bibr11-13634615231187257] Black E. B. Ranmuthugala G. Kondalsamy-Chennakesavan S. Toombs M. R. Nicholson G. C. Kisely S. (2015). A systematic review: Identifying the prevalence rates of psychiatric disorder in Australia’s indigenous populations. Australian & New Zealand Journal of Psychiatry, 49(5), 412–429. 10.1177/0004867415569802 25690747

[bibr12-13634615231187257] Bowen A. Duncan V. Peacock S. Bowen R. Schwartz L. Campbell D. Muhajarine N. (2014). Mood and anxiety problems in perinatal Indigenous women in Australia, New Zealand, Canada, and the United States: A critical review of the literature. Transcultural Psychiatry, 51(1), 93–111. 10.1177/1363461513501712 24065605

[bibr13-13634615231187257] Brinckley M.-M. Calabria B. Walker J. Thurber K. A. Lovett R. (2021). Reliability, validity, and clinical utility of a culturally modified Kessler scale (MK-K5) in the Aboriginal and Torres Strait Islander population. BMC Public Health, 21(1), 1–15. 10.1186/s12889-021-11138-4 34112127 PMC8194217

[bibr14-13634615231187257] Brown A. D. H. Mentha R. Howard M. Rowley K. Reilly R. Paquet C. O’Dea K. (2016). Men, hearts and minds: Developing and piloting culturally specific psychometric tools assessing psychosocial stress and depression in central Australian Aboriginal men. Social Psychiatry and Psychiatric Epidemiology, 51(2), 211–223. 10.1007/s00127-015-1100-8 26233468

[bibr15-13634615231187257] Brown A. D. H. Mentha R. Rowley K. G. Skinner T. Davy C. O'Dea K. (2013). Depression in Aboriginal men in central Australia: Adaptation of the Patient Health Questionnaire 9. BMC Psychiatry, 13, 10.1186/1471-244X-13-271 PMC3816593 24139186

[bibr16-13634615231187257] Brown A. Scales U. Beever W. Rickards B. Rowley K. O’Dea K. (2012). Exploring the expression of depression and distress in aboriginal men in central Australia: A qualitative study. BMC Psychiatry, 12(1), 97. 10.1186/1471-244X-12-97 22853622 PMC3441213

[bibr100-13634615231187257] Butler, T., Allnutt, S., Kariminia, A., & Cain, D. (2007). Mental health status of Aboriginal and non-Aboriginal Australian prisoners. *Australian & New Zealand Journal of Psychiatry*, *41*(5), 429–435. 10.1080/00048670701261210 17464735

[bibr17-13634615231187257] Cairney J. Veldhuizen S. Wade T. J. Kurdyak P. Streiner D. L. (2007). Evaluation of 2 measures of psychological distress as screeners for depression in the general population. The Canadian Journal of Psychiatry, 52(2), 111–120. Retrieved https://journals.sagepub.com/doi/pdf/10.1177/070674370705200209 10.1177/070674370705200209 17375867

[bibr18-13634615231187257] Carlin E. Atkinson D. Marley J. V. (2019). Having a quiet word: Yarning with aboriginal women in the pilbara region of Western Australia about mental health and mental health screening during the perinatal period. International Journal of Environmental Research and Public Health, 16(21). 10.3390/ijerph16214253 PMC6862568 31683908

[bibr19-13634615231187257] CDC. (2019–2022). *Summary health statistics* . Retrieved https://wwwn.cdc.gov/NHISDataQueryTool/SHS_adult/index.html

[bibr20-13634615231187257] Chan A. W. Reid C. Skeffington P. Marriott R. (2021). A systematic review of EPDS cultural suitability with Indigenous mothers: A global perspective. Archives of Women's Mental Health, 24(3), 353–365. 10.1007/s00737-020-01084-2 PMC8116293 33245435

[bibr21-13634615231187257] Comstock G. W. Helsing K. J. (1977). Symptoms of depression in two communities. Psychological Medicine, 6(4), 551–563. 10.1017/S0033291700018171 1005571

[bibr22-13634615231187257] Daniel M. Cargo M. D. Lifshay J. Green L. W. (2004). Cigarette smoking, mental health and social support. Canadian Journal of Public Health, 95(1), 45–49. 10.1007/BF03403633 14768741 PMC6975667

[bibr23-13634615231187257] Danyluck C. Blair I. V. Manson S. M. Laudenslager M. L. Daugherty S. L. Jiang L. Brondolo E. (2021). Older and wiser? Age moderates the association between discrimination and depressive symptoms in American Indians and Alaska natives. Journal of Aging & Health, 10S–17S. 10.1177/08982643211013699 34167343 PMC9087640

[bibr24-13634615231187257] Davis T. M. E. Hunt K. Bruce D. G. Starkstein S. Skinner T. McAullay D. Davis W. A. (2015). Prevalence of depression and its associations with cardio-metabolic control in Aboriginal and Anglo-Celt patients with type 2 diabetes: The Fremantle Diabetes Study Phase II. Diabetes Research and Clinical Practice, 107(3), 384–391. 10.1016/j.diabres.2014.12.014 25656760

[bibr25-13634615231187257] De Coteau T. J. Hope D. A. Anderson J. (2003). Anxiety, stress, and health in Northern Plains Native Americans. Behavior Therapy, 34(3), 365–380. https://doi.org/https://dx.https://doi.org/10.1016/S0005-7894%2803%2980006-0

[bibr26-13634615231187257] Dingwall K. M. Cairney S. (2010). Psychological and cognitive assessment of Indigenous Australians. Australian & New Zealand Journal of Psychiatry, 44(1), 20–30. 10.3109/00048670903393670 20073564

[bibr27-13634615231187257] Duran B. Sanders M. Skipper B. Waitzkin H. Malcoe L. H. Paine S. Yager J. (2004). Prevalence and correlates of mental disorders among Native American women in primary care. American Journal of Public Health, 94(1), 71–77. 10.2105/AJPH.94.1.71 14713701 PMC1449829

[bibr28-13634615231187257] Easton S. D. Roh S. Kong J. Lee Y.-S. (2019). Childhood sexual abuse and depression among American Indians in adulthood. Health & Social Work, 44(2), 95–103. 10.1093/hsw/hlz005 30809642

[bibr29-13634615231187257] Esler D. M. Johnston F. Thomas D. (2007). The acceptability of a depression screening tool in an urban, Aboriginal community-controlled health service. Australian and New Zealand Journal of Public Health, 31(3), 259–263. 10.1111/j.1467-842X.2007.00058.x 17679245

[bibr30-13634615231187257] Esler D. M. Johnston F. Thomas D. Davis B. (2008). The validity of a depression screening tool modified for use with Aboriginal and Torres Strait Islander people. Australian and New Zealand Journal of Public Health, 32(4), 317–321. 10.1111/j.1753-6405.2008.00247.x 18782392

[bibr31-13634615231187257] Evans-Campbell T. Walters K. L. Pearson C. R. Campbell C. D. (2012). Indian boarding school experience, substance use, and mental health among urban two-spirit American Indian/Alaska Natives. The American Journal of Drug and Alcohol Abuse, 38(5), 421–427. 10.3109/00952990.2012.701358 22931076 PMC5446670

[bibr32-13634615231187257] Faruk M. O. Nijhum R. P. Khatun M. N. Powell G. E. (2021). Anxiety and depression in two indigenous communities in Bangladesh. Global Mental Health, 8, 8. 10.1017/gmh.2021.33 PMC8411373 34527262

[bibr33-13634615231187257] Firestone M. Smylie J. Maracle S. McKnight C. Spiller M. O’Campo P. (2015). Mental health and substance use in an urban First Nations population in Hamilton, Ontario. Canadian Journal of Public Health, 106(6), e375–e381. 10.17269/CJPH.106.4923 PMC6972211 26680428

[bibr34-13634615231187257] FNDGC. (2018). *National report of the First Nations Regional Health Survey* . Retrieved https://fnigc.ca/wpcontent/uploads/2020/09/713c8fd606a8eeb021debc927332938d_FNIGC-RHS-Phase-III-Report1-FINAL-VERSION-Dec.2018.pdf

[bibr35-13634615231187257] Froese C. L. Butt A. Mulgrew A. Cheema R. Speirs M.-A. Gosnell C. Fleming J. Fleetham J. Frank Ryan C. Ayas N. T. (2008). Depression and sleep-related symptoms in an adult, indigenous, North American population. Journal of Clinical Sleep Medicine, 4(4), 356–361. Retrieved https://www.ncbi.nlm.nih.gov/pmc/articles/PMC2542493/pdf/jcsm.4.4.356.pdf 10.5664/jcsm.27237 18763428 PMC2542493

[bibr36-13634615231187257] Gachupin F. Romero M. D. Ortega W. J. Jojola R. Hendrie H. Torres E. P. Sr. Lujan F. Lente M. Sanchez B. Teller V. Beita F. Albeita U. Lente B. Gustafson D. R. (2016). Cognition, depressive symptoms and vascular factors among southwest tribal elders. Ethnicity & Disease, 26(2), 235–244. 10.18865/ed.26.2.235 27103775 PMC4836905

[bibr37-13634615231187257] Gall A. Anderson K. Howard K. Diaz A. King A. Willing E. Connolly M. Lindsay D. Garvey G. (2021). Wellbeing of Indigenous Peoples in Canada, Aotearoa (New Zealand) and the United States: A Systematic Review. International Journal of Environmental Research and Public Health, 18(11). 10.3390/ijerph18115832 PMC8198891 34071636

[bibr38-13634615231187257] Gallardo-Peralta L. P. Rodríguez-Blázquez C. Ayala-García A. Forjaz M. J. (2020). Multi-ethnic validation of 15-item Geriatric Depression Scale in Chile. Psicologia: Reflexão e Crítica, 33(1), 10.1186/s41155-020-00146-9 PMC7237627 32430560

[bibr39-13634615231187257] Gallardo-Peralta L. P. Sánchez-Moreno E. De Roda A. B. L. Astray A. A. (2015). Ethnicity, social support, and depression among elderly Chilean people. The Journal of Psychology: Interdisciplinary and Applied, 149(6), 601–629. 10.1080/00223980.2014.946462 25175789

[bibr40-13634615231187257] Getting it Right Collaborative G. (2019). Getting it Right: Validating a culturally specific screening tool for depression (aPHQ-9) in Aboriginal and Torres Strait Islander Australians. The Medical Journal of Australia, 211(1), 24–30. https://doi.org/https://dx.https://doi.org/10.5694/mja2.50212 31256439 10.5694/mja2.50212

[bibr41-13634615231187257] Goldwasser H. D. Badger L. W. (1989). Utility of the psychiatric screen among the Navajo of Chinle: A fourth-year clerkship experience. American Indian and Alaska Native Mental Health Research, 3(1), 6–15. 10.5820/aian.0301.1989.6 2490223

[bibr42-13634615231187257] Gomez Cardona L. Brown K. Goodleaf T. McComber M. D'Amico R. Phillips A. Boyer C. Martin C. Splicer B. Goodleaf S. Thompson D. Haswell M. Laliberté A. Linnaranta O. (2021). Cultural adaptation of an appropriate tool for mental health among Kanien'keha:ka: a participatory action project based on the Growth and Empowerment Measure. Social Psychiatry and Psychiatric Epidemiology. 10.1007/s00127-021-02164-z 34398264

[bibr43-13634615231187257] Gone J. P. Kirmayer L. J. (2020). Advancing indigenous mental health research: Ethical, conceptual and methodological challenges. Transcultural Psychiatry, 57(2), 235–249. 10.1177/1363461520923151 32380932

[bibr44-13634615231187257] Gray J. S. Brionez J. Petros T. Gonzaga K. T. (2019). Psychometric evaluation of depression measures with Northern Plains Indians. American Journal of Orthopsychiatry, 89(4), 534–541. 10.1037/ort0000309 29345480

[bibr45-13634615231187257] Gray J. S. McCullagh J. A. Petros T. (2016). Assessment of anxiety among Northern Plains Indians. The American Journal of Orthopsychiatry, 86(2), 186–193. https://doi.org/https://dx.https://doi.org/10.1037/ort0000103 26594919 10.1037/ort0000103

[bibr46-13634615231187257] Haggarty J. Cernovsky Z. Kermeen P. Merskey H. (2000). Psychiatric disorders in an Arctic community. The Canadian Journal of Psychiatry, 45(4), 357–362. Retrieved https://journals.sagepub.com/doi/pdf/10.1177/070674370004500404 10.1177/070674370004500404 10813069

[bibr47-13634615231187257] He J. van de Vijver F. (2012). Bias and equivalence in cross-cultural research. Online Readings in Psychology and Culture, 2(2), 2307–0919. 10.9707/2307-0919.1111

[bibr48-13634615231187257] Hsieh Y.-P. Roh S. Lee Y.-S. (2020). Spiritual well-being, social support, and depression among American Indian women cancer survivors: The mediating effect of perceived quality of life. Families in Society, 101(1), 83–94. 10.1177/1044389419853113

[bibr49-13634615231187257] Ing C. T. Antonio M. Ahn H. J. Cassel K. Dillard A. Kekauoha B. P. Kaholokula J. K. (2019). An examination of the relationship between discrimination, depression, and hypertension in native Hawaiians. Asian American Journal of Psychology, 10(3), 249–257. https://doi.org/https://dx.https://doi.org/10.1037/aap0000151 33224437 10.1037/aap0000151 PMC7678754

[bibr50-13634615231187257] Isaacs A. Lampitt B. (2014). The Koorie Men’s Health Day: An innovative model for early detection of mental illness among rural Aboriginal men. Australasian Psychiatry, 22(1), 56–61. 10.1177/1039856213502241 23996792

[bibr51-13634615231187257] Janca A. Lyons Z. Balaratnasingam S. Parfitt D. Davison S. Laugharne J. (2015). Here and Now Aboriginal Assessment: Background, development and preliminary evaluation of a culturally appropriate screening tool. Australasian Psychiatry, 23(3), 287–292. 10.1177/1039856215584514 25944764

[bibr52-13634615231187257] Jorm A. F. Bourchier S. J. Cvetkovski S. Stewart G. (2012). Mental health of indigenous Australians: A review of findings from community surveys. Medical Journal of Australia, 196(2), 118–121. 10.5694/mja11.10041 22304605

[bibr53-13634615231187257] Kading M. L. Hautala D. S. Palombi L. C. Aronson B. D. Smith R. C. Walls M. L. (2015). Flourishing: American Indian positive mental health. Society and Mental Health, 5(3), 203–217. 10.1177/2156869315570480 28966866 PMC5619867

[bibr54-13634615231187257] Kaholokula J. K. Grandinetti A. Crabbe K. M. Healani M. Chang K. Kenui C. K. (1999). Depressive symptoms and cigarette smoking among native hawaiians. Asia-Pacific Journal of Public Health, 11(2), 60–64. https://doi.org/https://dx.https://doi.org/10.1177/101053959901100202 11195159 10.1177/101053959901100202

[bibr55-13634615231187257] Kisely S. Alichniewicz K. K. Black E. B. Siskind D. Spurling G. Toombs M. (2017). The prevalence of depression and anxiety disorders in indigenous people of the Americas: A systematic review and meta-analysis. Journal of Psychiatric Research, 84, 137–152. 10.1016/j.jpsychires.2016.09.032 27741502

[bibr56-13634615231187257] Kohrt B. A. Rasmussen A. Kaiser B. N. Haroz E. E. Maharjan S. M. Mutamba B. B. de Jong J. T. Hinton D. E. (2014). Cultural concepts of distress and psychiatric disorders: Literature review and research recommendations for global mental health epidemiology. International Journal of Epidemiology, 43(2), 365–406. Retrieved https://www.ncbi.nlm.nih.gov/pmc/articles/PMC3997373/pdf/dyt227.pdf 10.1093/ije/dyt227 24366490 PMC3997373

[bibr57-13634615231187257] Kroenke K. (2021). PHQ-9: Global uptake of a depression scale. World Psychiatry, 20(1), 135–136. 10.1002/wps.20821 33432739 PMC7801833

[bibr58-13634615231187257] Kroenke K. Spitzer R. L. Williams J. B. W. (2001). The PHQ-9: Validity of a brief depression severity measure. Journal of General Internal Medicine, 16(9), 606–613. 10.1046/j.1525-1497.2001.016009606.x 11556941 PMC1495268

[bibr59-13634615231187257] Le Grande M. Ski C. F. Thompson D. R. Scuffham P. Kularatna S. Jackson A. C. Brown A. (2017). Social and emotional wellbeing assessment instruments for use with Indigenous Australians: A critical review. Social Science & Medicine, 187, 164–173. 10.1016/j.socscimed.2017.06.046 28689090

[bibr60-13634615231187257] Lee C. Wozniak L. A. Soprovich A. L. Sharma V. Healy B. Samanani S. Eurich D. T. (2022). Mental health experiences with COVID-19 public health measures in an Alberta First Nations Community. International Journal of Mental Health Systems, 16(1), 22. https://doi.org/https://dx.https://doi.org/10.1186/s13033-022-00532-z 35488309 10.1186/s13033-022-00532-z PMC9051493

[bibr61-13634615231187257] Letiecq B. L. Bailey S. J. Kurtz M. A. (2008). Depression among rural Native American and European American grandparents rearing their grandchildren. Journal of Family Issues, 29(3), 334–356. 10.1177/0192513(07308393

[bibr62-13634615231187257] MacMillan H. L. Jamieson E. Walsh C. A. Wong M. Y. Y. Faries E. J. McCue H. MacMillan A. B. Offord D. R. , & with The Technical Advisory Committee of the Chiefs of Ontario. (2008). First nations women's mental health: Results from an Ontario survey. Archives of Women's Mental Health, 11(2), 109–115. https://doi.org/https://dx.https://doi.org/10.1007/s00737-008-0004-y 10.1007/s00737-008-0004-y 18493709

[bibr63-13634615231187257] The MAGPiE Group (2005). Mental disorders among Maori attending their general practitioner. Australian & New Zealand Journal of Psychiatry, 39(5), 401–406. 10.1111/j.1440-1614.2005.01588.x 15860029

[bibr64-13634615231187257] Montag A. C. Brodine S. K. Alcaraz J. E. Clapp J. D. Allison M. A. Calac D. J. Hull A. D. Gorman J. R. Jones K. L. Chambers C. D. (2015). Effect of depression on risky drinking and response to a screening, brief intervention, and referral to treatment intervention. American Journal of Public Health, 105(8), 1572–1576. https://doi.org/https://dx.https://doi.org/10.2105/AJPH.2015.302688 26066915 10.2105/AJPH.2015.302688 PMC4504277

[bibr65-13634615231187257] Nelson S. E. Wilson K. (2017). The mental health of Indigenous peoples in Canada: A critical review of research. Social Science & Medicine, 176, 93–112. 10.1016/j.socscimed.2017.01.021 28135694

[bibr66-13634615231187257] Northern Norway. (n.d.). *The Sami* . Retrieved https://nordnorge.com/en/tema/the-sami-are-the-indigenous-people-of-the-north/

[bibr67-13634615231187257] Page, M. J., McKenzie, J. E., Bossuyt, P. M., Boutron, I., Hoffmann, T. C., Mulrow, C. D., Shamseer, L., Tetzlaff, J. M., Akl, E. A., Brennan, S. E., & Chou, R. (2021). The PRISMA 2020 statement: An updated guideline for reporting systematic reviews. *International Journal of Surgery*, *88*, 105906.

[bibr68-13634615231187257] Peters M. D. J. (2017). Managing and coding references for systematic reviews and scoping reviews in EndNote. Medical Reference Services Quarterly, 36(1), 19–31. 10.1080/02763869.2017.1259891 28112629

[bibr69-13634615231187257] Peters, M. D. J., Godfrey, C., McInerney, P., Munn, Z., Tricco, A. C., & Khalil, H. Chapter 11: Scoping Reviews (2020 version). In: Aromataris E. & Munn Z. (Eds.). *JBI Manual for Evidence Synthesis*, JBI, 2020. Retreived https://synthesismanual.jbi.global. 10.46658/JBIMES-20-12

[bibr70-13634615231187257] PRISMA. (2020). *Preferred Reporting Items for Systematic Reviews and Meta-analyses extension for scoping review (PRISMA-ScR) flow diagram* . Retrieved http://www.prisma-statement.org/PRISMAStatement/FlowDiagram

[bibr71-13634615231187257] Radloff L. S. (1977). The CES-D scale: A self-report depression scale for research in the general population. Applied Psychological Measurement, 1(3), 385–401. 10.1177/014662167700100306

[bibr72-13634615231187257] Reynolds C. R. Suzuki L. A. (2012). Bias in psychological assessment: An empirical review and recommendations. Handbook of Psychology, Second Edition, 10. 10.1002/9781118133880.hop210004

[bibr73-13634615231187257] Roberts, R. E., & Vernon, S. W. (1983). The Center for Epidemiological Studies Depression Scale: Its use in a community sample. *The American Journal of Psychiatry*, *140*(1), 41–46. 10.1176/ajp.140.1.41

[bibr74-13634615231187257] Roh S. Burnette C. E. Lee K. H. Lee Y.-S. Easton S. D. Lawler M. J. (2015). Risk and protective factors for depressive symptoms among American Indian older adults: Adverse childhood experiences and social support. Aging & Mental Health, 19(4), 371–380. https://doi.org/https://dx.https://doi.org/10.1080/13607863.2014.938603 25070293 10.1080/13607863.2014.938603

[bibr75-13634615231187257] Russell S. G. Quigley R. Thompson F. Sagigi B. LoGiudice D. Smith K. Pachana N. Miller G. Strivens E. (2021). Prevalence of dementia in the Torres Strait. Australasian Journal on Ageing, 40(2), e125–e132. 10.1111/ajag.12878 33169520

[bibr76-13634615231187257] Russell S. G. Quigley R. Thompson F. Sagigi B. Miller G. LoGiudice D. Smith K. Strivens E. Pachana N. A. (2022). Culturally appropriate assessment of depression and anxiety in older Torres Strait Islanders: Limitations and recommendations. Clinical Gerontologist, 46(2), 240–252. 10.1080/07317115.2022.2086090 35694996

[bibr77-13634615231187257] Sahota P. K. C. Knowler W. C. Looker H. C. (2008). Depression, diabetes, and glycemic control in an American Indian community. The Journal of Clinical Psychiatry, 69(5), 800–809. 10.4088/JCP.v69n0513 18370573 PMC2574858

[bibr78-13634615231187257] Shen Y.-T. Radford K. Daylight G. Cumming R. Broe T. G. A. Draper B. (2018). Depression, suicidal behaviour, and mental disorders in older Aboriginal Australians. International Journal of Environmental Research and Public Health, 15(3), 447. 10.3390/ijerph15030447 29510527 PMC5876992

[bibr79-13634615231187257] Shore J. H. Manson S. M. (1981). Cross-cultural studies of depression among American Indians and Alaska Natives. White Cloud Journal of American Indian/Alaska Native Mental Health, 2(2), 5–12.

[bibr80-13634615231187257] Snodgrass J. G. Lacy M. G. Upadhyay C. (2017). Developing culturally sensitive affect scales for global mental health research and practice: Emotional balance, not named syndromes, in Indian Adivasi subjective well-being. Social Science and Medicine, 187, 174–183. 10.1016/j.socscimed.2017.06.037 28704701

[bibr81-13634615231187257] Sorlie T. Hansen K. L. Friborg O. (2018). Do Norwegian sami and non-indigenous individuals understand questions about mental health similarly? A SAMINOR 2 study. International Journal of Circumpolar Health, 77(1), 1481325. https://doi.org/https://dx.https://doi.org/10.1080/22423982.2018.1481325 29869591 10.1080/22423982.2018.1481325 PMC5990933

[bibr82-13634615231187257] Sørly R. Mathisen V. Kvernmo S. (2021). We belong to nature: Communicating mental health in an indigenous context. Qualitative Social Work, 20(5), 1280–1296. 10.1177/1473325020932374

[bibr83-13634615231187257] Taylor S. McDermott R. Thompson F. Usher K. (2017). Depression and diabetes in the remote Torres Strait Islands. Health Promotion Journal of Australia, 28(1), 59–66. Retrieved https://onlinelibrary.wiley.com/doi/abs/10.1071/HE15118 10.1071/HE15118 27464880

[bibr84-13634615231187257] Tu D. Hadjipavlou G. Dehoney J. Price E. R. Dusdal C. Browne A. J. Varcoe C. (2019). Partnering with Indigenous Elders in primary care improves mental health outcomes of inner-city Indigenous patients: Prospective cohort study. Canadian Family Physician Medecin de Famille Canadien, 65(4), 274–281. 30979762 PMC6467659

[bibr85-13634615231187257] United Nations. (2007). *United Nations Declaration on the Rights of Indigenous Peoples* . Retrieved https://www.un.org/development/desa/indigenouspeoples/wp-content/uploads/sites/19/2018/11/UNDRIP_E_web.pdf

[bibr86-13634615231187257] United Nations. (2013). *State of the world's indigenous peoples. Indigenous peoples’ access to health services* (Vol. 2, p. 190). Retrieved https://www.un.org/development/desa/indigenouspeoples/wp-content/uploads/sites/19/2018/03/The-State-of-The-Worlds-Indigenous-Peoples-WEB.pdf

[bibr87-13634615231187257] United Nations. (n.d). *Indigenous peoples, indigneous voices fact sheet* . Retrieved https://www.un.org/esa/socdev/unpfii/documents/5session_factsheet1.pdf

[bibr88-13634615231187257] Verney S. P. Jervis L. L. Fickenscher A. Roubideaux Y. Bogart A. Goldberg J. (2008). Symptoms of depression and cognitive functioning in older American Indians. Aging & Mental Health, 12(1), 108–115. https://doi.org/https://dx.https://doi.org/10.1080/13607860701529957 18297485 10.1080/13607860701529957

[bibr89-13634615231187257] Walker R. J. Campbell J. A. Dawson A. Z. Egede L. E. (2019). Prevalence of psychological distress, depression and suicidal ideation in an indigenous population in Panamá. Social Psychiatry & Psychiatric Epidemiology, 54(10), 1199–1207. 10.1007/s00127-019-01719-5 31055631 PMC6790172

[bibr90-13634615231187257] Walls M. Pearson C. Kading M. Teyra C. (2016). Psychological wellbeing in the face of adversity among American Indians: Preliminary evidence of a new population health paradox? Annals of Public Health and Research, 3(1), 1034. 28553671 PMC5443649

[bibr91-13634615231187257] Warne D. Dulacki K. Spurlock M. Meath T. Davis M. M. Wright B. McConnell K. J. (2017). Adverse Childhood Experiences (ACE) among American Indians in South Dakota and Associations with mental health conditions, alcohol use, and smoking. Journal of Health Care for the Poor & Underserved, 28(4), 1559–1577. 10.1353/hpu.2017.0133 29176114

[bibr92-13634615231187257] Westermeyer J. Neider J. (1984). Depressive symptoms among native American alcoholics at the time of a 10-year follow-up. Alcoholism: Clinical and Experimental Research, 8(5), 429–434. 10.1111/j.1530-0277.1984.tb05696.x 6391251

[bibr101-13634615231187257] Whitbeck, L. B., McMorris, B. J., Hoyt, D. R., Stubben, J. D., & LaFromboise, T. (2002). Perceived discrimination, traditional practices, and depressive symptoms among American Indians in the upper Midwest. *Journal of Health and Social Behavior*, 400–418. 12664673

[bibr93-13634615231187257] Whitbeck L. B. Hoyt D. Johnson K. Chen X. (2006). Mental disorders among parents/caretakers of American Indian early adolescents in the Northern Midwest. Social Psychiatry and Psychiatric Epidemiology, 41(8), 632–640. 10.1007/s00127-006-0070-2 16779502 PMC2593415

[bibr94-13634615231187257] Williamson A. B. D'Este C. A. Clapham K. F. Eades S. J. Redman S. Raphael B. (2016). Psychological distress in carers of Aboriginal children in urban New South Wales: Findings from SEARCH (phase one). Medical Journal of Australia, 205(1), 27–32. 10.5694/mja16.00111 27362684

[bibr95-13634615231187257] Wilson C. Civic D. Glass D. (1995). Prevalence and correlates of depressive syndromes among adults visiting an Indian Health Service primary care clinic. American Indian and Alaska Native Mental Health Research, 6(2), 1–12. 10.5820/aian.0602.1995.1 7734607

[bibr96-13634615231187257] Wilson K. Richmond C. (2009). Indigenous health and medicine. In: Kitchin R. Thrift N. (Eds.), International encyclopedia of human geography (pp. 365–370). Elsevier.

[bibr97-13634615231187257] Wong A. Hyde Z. Smith K. Flicker L. Atkinson D. Skeaf L. Malay R. LoGiudice D. (2021). Prevalence and sites of pain in remote-living older aboriginal Australians, and associations with depressive symptoms and disability. Internal Medicine Journal, 51(7), 1092–1100. 10.1111/imj.14870 32359117

