# Supplementary material for: Screening depression and anxiety in Indigenous peoples: A global scoping review
Source: Transcult Psychiatry. 2023 Jul 25;62(2):289–304. doi: 10.1177/13634615231187257 (PMC12130600; doi:10.1177/13634615231187257)
Supplement: sj-pdf-1-tps-10.1177_13634615231187257 - Supplemental material for Screening depression and anxiety in Indigenous peoples: A global scoping review [file sj-pdf-1-tps-10.1177_13634615231187257.pdf]

Supplementary file 1

Scoping review database searches

All databases search between 26 August – 4 October 2022

Note – not all search strings are the same due to differences in each database.

Ovid MEDLINE(R) and Epub Ahead of Print, In-Process, In-Data-Review & Other Non-Indexed Citations, Daily and Versions <1946 to August 31, 2022>

((“Indigenous peoples”[Mesh] OR "Native Hawaiian or Other Pacific Islander" [Mesh] OR exp American Native Continental Ancestry Group [Mesh])) OR (((indigenous peoples OR first nations OR first peoples OR aboriginal OR indigenous OR native american OR native Hawaiin OR american indian OR maori OR inuit OR alskan native OR metis OR australian aboriginal OR torres strait islander OR sami OR adavasi OR canadian aboriginal))

AND

“depression” [Mesh] OR “anxiety” [Mesh] OR anxiety OR depression

AND

tool* OR measur* OR screen* OR evaluat*

AND

Limit to “English language”

AND

Limit to ("young adult (19 to 24 years)" or "adult (19 to 44 years)" or "middle aged (45 plus years)" or "all aged (65 and over)" or "aged (80 and over)"))

PubMed

(("Indigenous peoples"[Mesh] OR "Native Hawaiian or Other Pacific Islander"[Mesh] OR ((("indigenous peoples" OR "first nations" OR "first peoples" OR aboriginal OR indigenous OR "native american" OR "native Hawaiin" OR "american indian" OR maori OR inuit OR "alskan native" OR metis OR "australian aboriginal" OR "torres strait islander" OR sami OR adavasi OR "canadian aboriginal"))

AND

depression[Mesh] OR anxiety[Mesh] OR anxiety OR depression

AND

tool* OR measur* OR screen* OR evaluat*

AND

"Limit to “English language”

NOT

“child OR adolescent OR school OR infant OR secondary school”

(("Indigenous peoples"[Mesh] OR "Native Hawaiian or Other Pacific Islander"[Mesh] OR ((("indigenous peoples" OR "first nations" OR "first peoples" OR aboriginal OR indigenous OR "native american" OR "native Hawaiin" OR "american indian" OR maori OR inuit OR "alskan native" OR metis OR "australian aboriginal" OR "torres strait islander" OR sami OR adavasi OR "canadian aboriginal")) AND depression[Mesh] OR anxiety[Mesh] OR anxiety OR depression AND tool* OR measur* OR screen* OR evaluat* ANDLimit to "English language" NOT "child* OR adolesc* OR school* OR infant* OR "secondary school*" - Schema: all

CINAHL

(MH "Native Americans") OR (MH "First Nations of Australia") OR (MH "Indigenous Peoples") OR (MH "Aboriginal Canadians") OR (MH "Aboriginal Australians") OR (MH "Torres Strait Islanders") OR (MH "First Nations of Canada") OR (MH "Maori") OR "indigenous peoples OR (Native Hawaiian or Other Pacific Islander) OR ("first nations" OR "first peoples" OR aboriginal OR indigenous OR "native american" OR "native Hawaiin" OR "american indian" OR maori OR inuit OR "alskan native" OR metis OR "australian aboriginal" OR "torres strait islander" OR sami OR adavasi OR "canadian aboriginal") AND depression OR anxiety AND (tool* OR measur* OR screen* OR evaluat*) NOT (child* OR adolesc* OR school* OR infant* OR secondary school*)"

PsychInfo

if(Indigenous Peoples) OR if(("first nations" OR "first peoples" OR aboriginal OR indigenous OR "Native American" OR "Native Hawaiian" OR "American Indian" OR Maori OR Inuit OR Alaskan Native OR "Aboriginal and Torres Strait Islander" OR Sami OR Adavasi OR Canadian Aboriginal)) AND if(depression) OR if(anxiety) AND if(screen* OR tool* OR measure* OR evaluat*) AND if(adult)

Ovid Emcare

Ovid Emcare <1995 to 2022 Week 34>

indigenous people/

(Native Hawaiian or Other Pacific Islander).mp. [mp=title, abstract, heading word, drug trade name, original title, device manufacturer, drug manufacturer, device trade name, keyword heading word]

exp American Indian/

(indigenous peoples or first nations or first peoples or aboriginal or indigenous or native american or native Hawaiin or american indian or maori or inuit or alskan native or metis or australian aboriginal or torres strait islander or sami or adavasi or canadian aboriginal).mp. [mp=title, abstract, heading word, drug trade name, original title, device manufacturer, drug manufacturer, device trade name, keyword heading word]

depression/

anxiety/

(depression or anxiety).mp. [mp=title, abstract, heading word, drug trade name, original title, device manufacturer, drug manufacturer, device trade name, keyword heading word]

(tool* or measur* or screen* or evaluat*).mp. [mp=title, abstract, heading word, drug trade name, original title, device manufacturer, drug manufacturer, device trade name, keyword heading word]

limit to English language

Scopus

(TITLE-ABS-KEY (*indigenous* AND *people*) OR TITLE-ABS-KEY (*native* AND *hawaiian* OR *other* AND *pacific* AND *islander*) OR TITLE-ABS-KEY (*indigenous* AND *peoples* OR *first* AND *nations* OR *first* AND *peoples* OR *aboriginal* OR *indigenous* OR *native* AND *american* OR *native* AND *hawaiin* OR *american* AND *indian* OR *maori* OR *inuit* OR *alskan* AND *native* OR *metis* OR *australian* AND *aboriginal* OR *torres* AND *strait* AND *islander* OR *sami* OR *adavasi* OR *canadian* AND *aboriginal*) AND TITLE-ABS-KEY (*depression*) OR TITLE-ABS-KEY (*anxiety*) AND TITLE-ABS-KEY (*tool** OR *measur** OR *screen** OR *evaluat**) AND TITLE-ABS-KEY (*adult*) AND NOT TITLE-ABS-KEY (*child** OR *adolesc** OR *school** OR *infant** OR *"secondary school*"*))
